# Radiation protection considerations for ultra-high dose rate proton beam FLASH research: consensus from multi-institutional experience

**DOI:** 10.3389/fonc.2026.1862653

**Published:** 2026-09-28

**Authors:** Michael J. Taylor, Christopher Bayer, Gerard Paquet, Nilendu Gupta, Michael A. Lamba, Haibo Lin, Sina Mossahebi, Yannick Poirier, Zhiyan Xiao, Francis Yu, Daniel R. Owen, Richard A. Amos, Wendy L. Smith

**Affiliations:** 1 Division of Cancer Sciences, University of Manchester and the Christie NHS Foundation Trust, Manchester, United Kingdom; 2 Varian, A Siemens Healthineers Company, Palo Alto, CA, United States; 3 Department of Radiation Oncology, The Ohio State University, Columbus, OH, United States; 4 Department of Radiation Oncology, University of Cincinnati College of Medicine, Cincinnati Children’s Hospital Medical Center, Cincinnati, OH, United States; 5 Medical Physics and Dosimetry, New York Proton Center, New York, NY, United States; 6 Department of Radiation Oncology, University of Maryland School of Medicine, Baltimore, MD, United States; 7 Department of Medical Physics and Biomedical Engineering, University College London, London, United Kingdom

**Keywords:** FLASH radiotherapy, neutron activation, proton beam therapy, radiation protection, ultra-high dose rate (UHDR)

## Abstract

The use of ultra-high dose rate (UHDR) radiation for radiotherapy could potentially improve outcomes for cancer patients by increasing healthy tissue sparing, known as the FLASH effect. As UHDR use expands in FLASH research and clinical translation, reassessing existing radiation safety programs is essential to determine whether UHDR beams introduce any new radiation safety requirements. This manuscript outlines the lessons learnt through investigations performed by members of the Varian FLASH forward consortium radiation protection working group. The information contained within is the consensus of the working group, as agreed, through discussions and reports during the monthly meetings. The paper brings together multi-institutional radiation protection experience from five Varian ProBeam type facilities engaged in preclinical and clinical proton FLASH research. Three main aspects of radiation protection are considered: shielding, activation, and accident scenarios. Lessons learned from the collective experience gained are presented to benefit those embarking on new proton FLASH research programs.

## 1 Introduction

An increase in healthy tissue sparing, the so-called FLASH effect, has been observed when a large (≥8 Gy) therapeutic radiation dose is delivered at an ultra-high dose rate (UHDR) of >40 Gy/s, compared to conventional radiotherapy (RT) dose rates of the order of 2 Gy/min (1). Due to the unique capability of cyclotron-based clinical proton beam therapy (PBT) systems to deliver doses at UHDR, much of the research effort into FLASH RT is performed in clinical PBT facilities. In order to achieve UHDR, proton FLASH research is often conducted using a single energy layer at the maximum allowable cyclotron beam current and energy, resulting in sufficient beam intensity at the nozzle. For Varian facilities, this is typically 800 nA cyclotron current at 250 MeV, although therapeutic dose rates exceeding 40 Gy/s have been observed at the center of a single spot for clinical 245 MeV deliveries.

The highest dose rate with PBT is achieved in the so-called entry plateau region of the Bragg curve, where spot sizes are at their narrowest, reducing the number of spots which contribute dose to a particular voxel and thus decreasing the “effective time” of radiation delivery as well as the instantaneous dose rate of each spot (2, 3). If a therapeutic dose is delivered in the plateau region, this precludes use of the Bragg peak which would deliver a much (double or more) higher dose outside of the target, therefore, the beam geometry is chosen to exit the body and safely deliver the Bragg peak outside the body — a geometry known as 'transmission' dose delivery as opposed to conventional delivery, in which the target is covered using a spread-out Bragg peak to minimize dose to normal tissues. It should be noted that past and ongoing human clinical trials for proton FLASH have so far used transmission beams (4–6).

Despite the larger spot size, there is growing interest in developing spread-out Bragg peak proton FLASH delivery techniques. To maintain sufficiently high beam intensities through the Varian ProBeam beam transport system, whilst achieving tumor targeting with a spread-out Bragg peak (SOBP), bespoke beam modulating devices (i.e. ridge filters) are used in the treatment room to control proton range and lateral penumbra of the high-energy beam (7). Such filters typically produce ~3 cm SOBP while maintaining UHDR conditions.

As interest in UHDR radiotherapy has grown, many centers have begun adapting existing clinical proton centers for UHDR delivery. Doing so requires operating at dose rates far beyond those for which current radiation safety systems were originally designed or evaluated. Higher dose rates inevitably increase the production of activated materials or secondary radiation in areas near the beam path. Consequently, a thorough reassessment of all components of the radiation safety program is essential to ensure safe operation under these elevated, dose and dose-rate, conditions.

This article presents considerations from the FlashForward Consortium Proton Radiation Safety Working Group for the enablement of UHDR FLASH delivery in research and clinical PBT facilities using the Varian ProBeam and ProBeam360° systems. Three room layouts are considered: ProBeam and ProBeam360° gantry rooms as well as a fixed horizontal beam research room. The principles presented are generic enough to apply to other cyclotron-based PBT facilities and are not specific to Varian proton delivery systems.

## 2 Methodology

This manuscript summarizes lessons learned from investigations conducted by members of the Varian FLASHForward Consortium Radiation Protection Working Group, which includes contributors with experience at five proton therapy centers: the University of Manchester (8), The Ohio State University, Cincinnati Children’s Hospital Medical Center, Emory Proton Therapy Center, and the University of Maryland. The manuscript content was developed through an informal consensus process based on discussion of institutional experience, reports presented during monthly working group meetings and written summaries reviewed by all participating members. Findings derived from measurements, simulations, or published literature are identified as such, whereas other observations reflect practical experience gained by working group members during proton FLASH radiation protection investigations. We emphasize that radiation protection considerations for proton FLASH radiotherapy are intrinsically site- and use-specific, therefore, the findings presented throughout this manuscript are intended to highlight areas that centers may wish to consider, rather than to define requirements or provide an exhaustive radiation safety program for proton FLASH research.

## 3 General radiation protection considerations

The safety and protection of staff, patients and the public are the primary motivating factors for any radiation protection program (9, 10). With the increased radiation dose and dose rate required for FLASH RT, secondary radiation dose and dose rate will be higher than for conventional RT deliveries. This work mainly focusses on radiation protection from secondary dose. The intended dose delivered to a target will be referred to as therapeutic dose.

In addition to total accumulated dose, some jurisdictions also regulate dose rate (instantaneous or over a short period of time, e.g. 20 µSv in any one hour in the US and 7.5 µSv/h in the UK) in certain circumstances. It will be incumbent on each program to review national and local regulations and ensure the provisions put in place are not only fit-for-purpose but also fall within those regulations. Another major consideration is occupancy levels. Areas of high occupancy with a high instantaneous dose rate may need to be controlled, whereas areas of high instantaneous dose rate but extremely low occupancy may not need to be controlled so tightly. The level of control would need to be guided by the legislation of the state hosting the center. Shielding, activation, and accident scenarios are typical areas for consideration when developing a radiation protection program.

The specific radiation protection measures required will very much depend on the geometry of the facilities used in UHDR delivery, as it dictates the distribution of secondary radiation and therefore radiation safety requirements. A fixed-beam room with a horizontal beam line configuration, such as that shown in **Figure 1**, is the simplest system with regard to implementing radiation protection measures due to the reduction in angular variance. In contrast, systems that utilize a gantry configuration – e.g. Varian’s ProBeam Gantry system (**Figure 2**) and Varian’s ProBeam360° gantry system (**Figure 3**) – must account for various angles of UHDR delivery.

**Figure 1:**
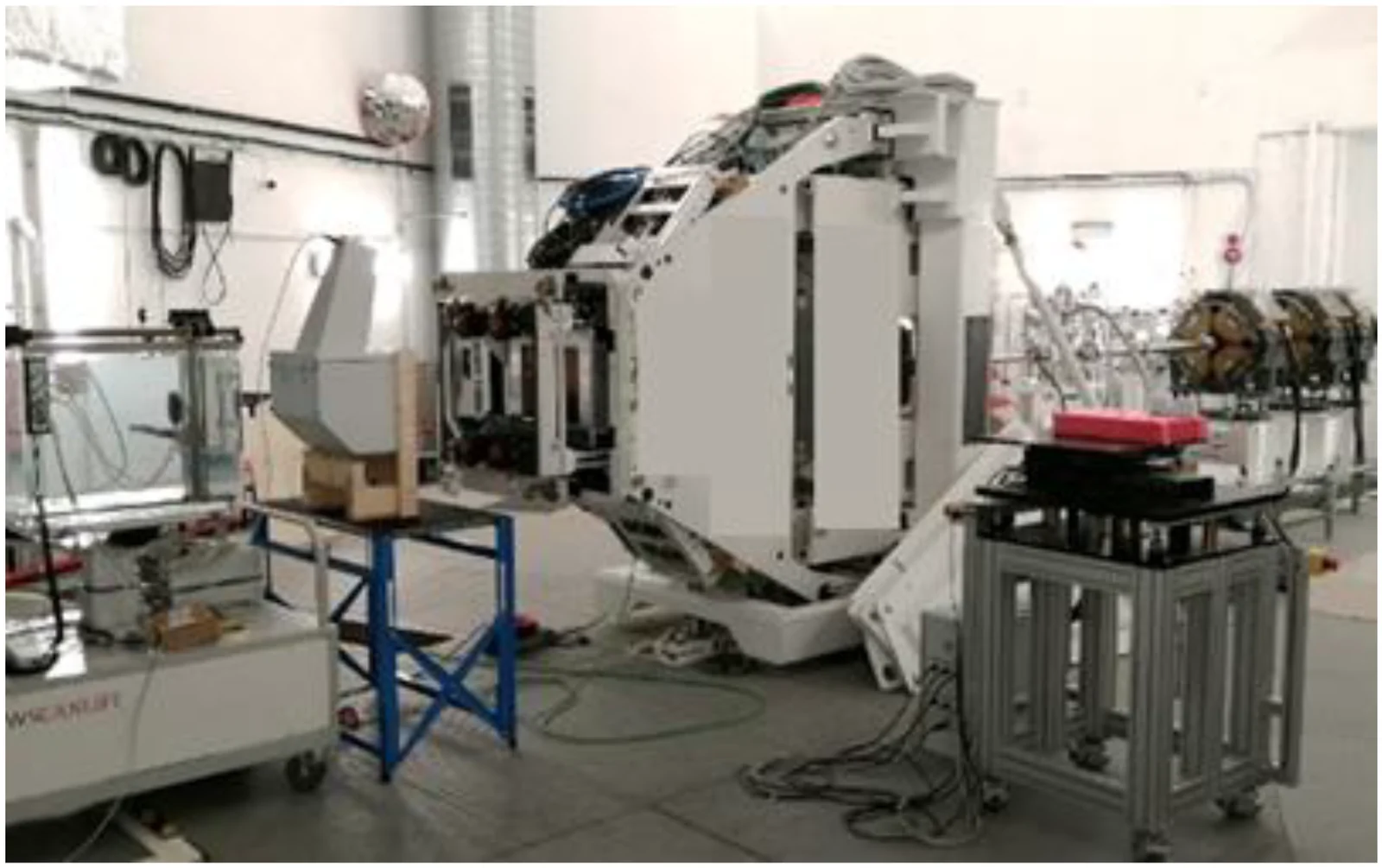
A photo of the Manchester fixed horizontal beam-line proton beam therapy research room.

**Figure 2:**
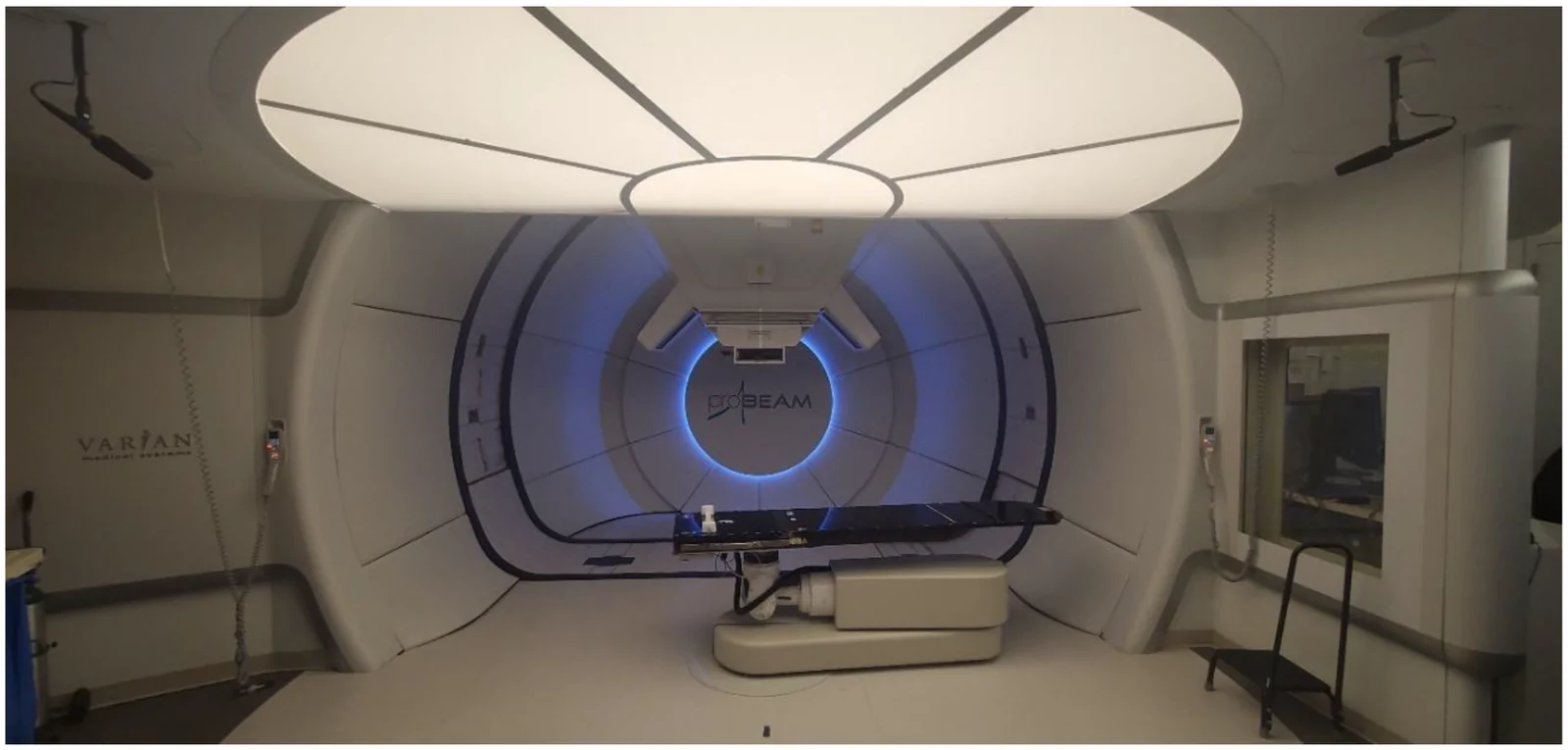
A photo of the Emory proton therapy center ProBeam gantry treatment room at 0 degrees.

**Figure 3:**
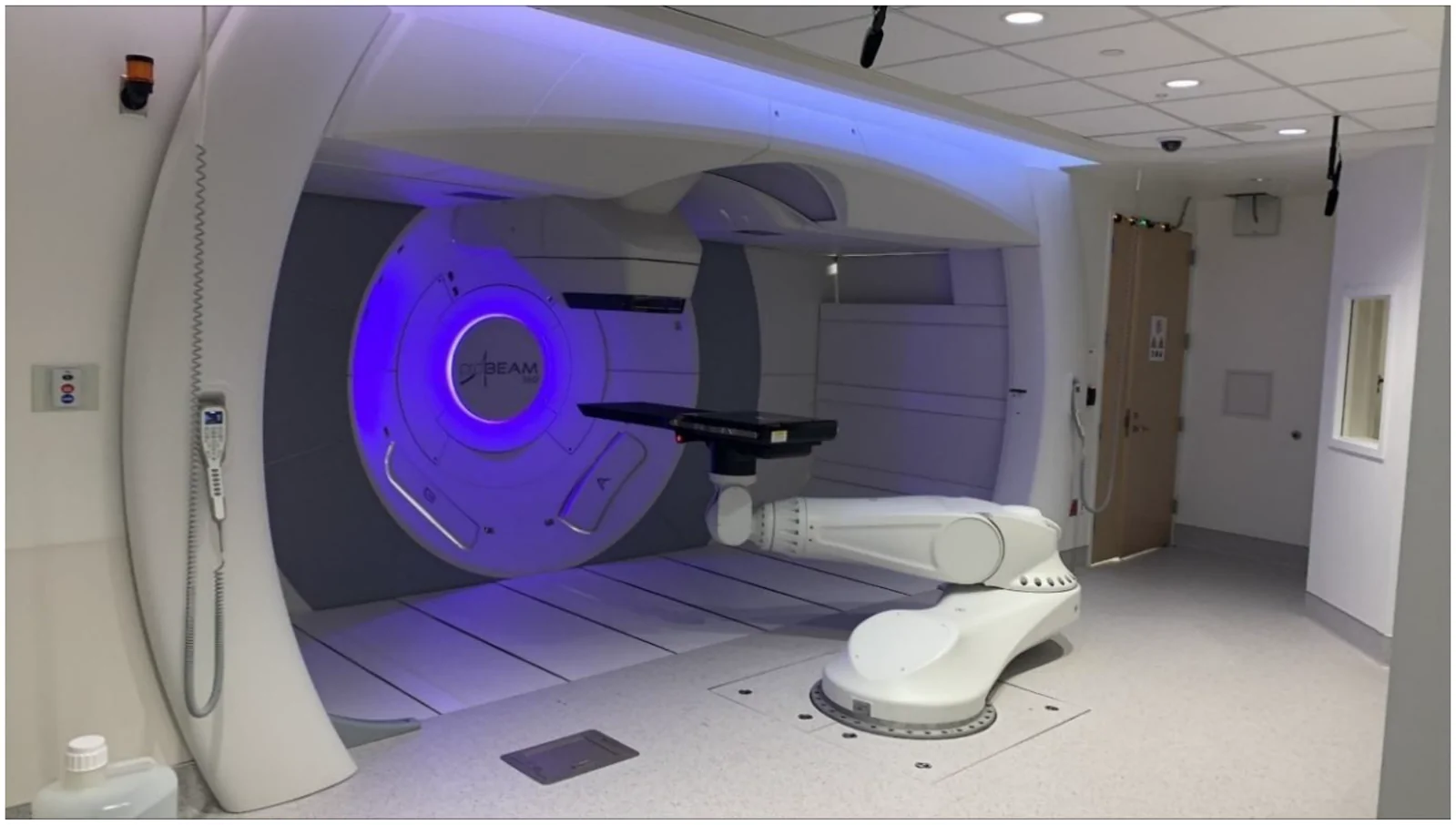
A photo of the Ohio State University comprehensive cancer center ProBeam360° gantry treatment room at 0 degrees.

The ProBeam and ProBeam360° configurations differ in many ways. The ProBeam gantry and fixed-beam room configurations utilize a maximum beam energy of 250 MeV, whereas the ProBeam360° is limited to 226 MeV. The ProBeam gantry system weighs about 180 tonnes (200 metric tons) and uses 45° and 135° bending magnets, along the beamline, for isocenter delivery, while the ProBeam360° gantry weighs about 72 tons (90 metric tons) and uses 50° and 150° bending magnets. The ProBeam360° gantry system is contained within a smaller footprint overall compared to the ProBeam gantry system. Both systems use an identical isochronous cyclotron (11).

## 4 Shielding provision

The key question in radiation shielding is whether the provisions employed at existing centers are sufficient for FLASH beam delivery. While empirical measurements are essential to definitively answer this, estimates for expected dose rates can be assessed prior to performing the full dose rate survey. Two estimation methods are described below.

### 4.1 Conventional dose rate radiation survey

Each center should estimate sources of exposure from UHDR beams prior to beginning UHDR irradiations. Radiation safety survey results for conventional deliveries (12) could be a useful starting point, as these results will provide an assessment of the radiation shielding and may already indicate “hot spots” (areas of high radiation exposure risk) which should be thoroughly investigated in a UHDR radiation survey (13, 14). This prior survey will **not solely** be sufficient to accurately predict all of the hot spots resulting from FLASH beam deliveries, as previous UHDR surveys have shown hotspots in new locations compared to conventional dose-rate surveys, but may at least highlight locations requiring specific attention (15). In addition, scaling from conventional (CONV) to UHDR beams is limited by the uncertainty surrounding low-value dose-rate measurements at conventional beam currents e.g. a value above background needs to be measured to scale for FLASH beam currents. It should also be noted that the angular distribution of the neutron field varies with proton beam energy, see **Figure 4**. As previously stated, achieving FLASH dose rates requires high proton beam energies to overcome the lack of beam transport system efficiency at lower beam energies, so this angular variability is of particular concern.

**Figure 4:**
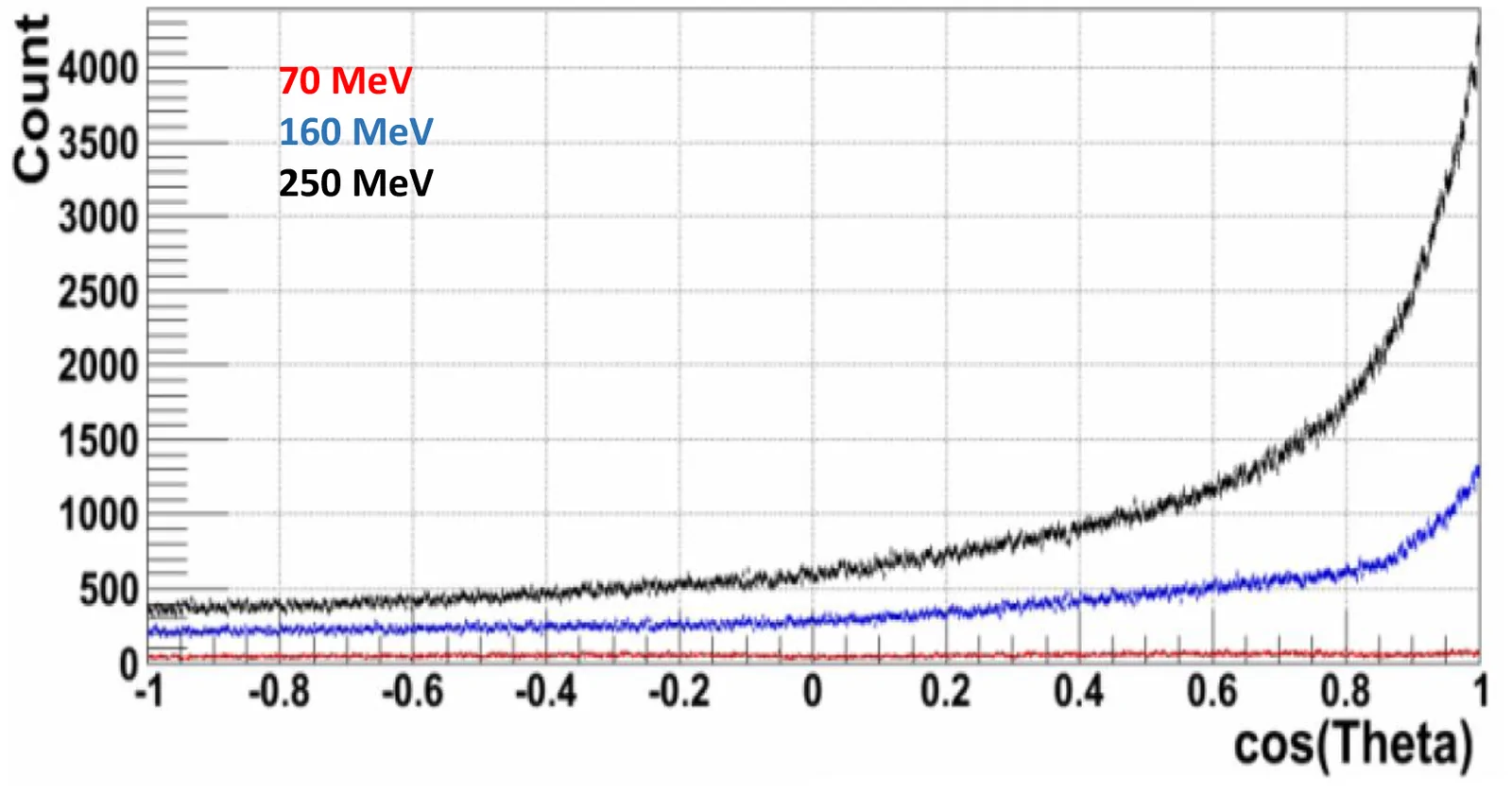
Results from a Monte-Carlo radiation transport simulation, using Geant4, for the angular distribution of neutrons emitted through proton-nuclear inelastic reactions for proton beam energies of 70 MeV (Red), 160 MeV (Blue) and 250 MeV (Black) impinging on a water plotting tank. There is a clear angular dependence on beam energy due to competing reactions with differing cross-sections.

### 4.2 Monte-Carlo radiation transport simulations

In addition to scaling measured data from conventional dose rate surveys, Monte-Carlo (MC) radiation transport simulations can be used to assess whether existing shielding is adequate to perform a UHDR dose rate survey (16). As noted previously, scaling prior measurements in areas where the dose rate did not exceed background levels amounts to little more than scaling background noise, providing no meaningful insight for survey values. One might reason that a low reading during a conventional dose rate survey implies that similarly low values will be measured under UHDR conditions – a reasonable inference given that both modalities use the same beamline and shielding geometry.

By employing established codes such as MCNP (17), Geant4 (18) and Fluka (19), dose rates can be simulated at key high-occupancy locations, enabling an informed assessment of whether in-person UHDR dose rate surveys can be conducted safely. Where validated results confirm that certain locations are safe to occupy, the simulations can further provide estimates for areas that cannot be safely surveyed by staff. It should be noted, however, that all MC simulations results require validation through empirical measurement. The accuracy of predicted dose rates depends on precise knowledge of shielding composition, physical dimensions and geometry, as well as the selection of appropriate physics models. Once expected dose rates in candidate areas have been deemed to be safe through this process, a comprehensive survey of those areas can proceed.

MCNP simulations performed by Varian using a 250 MeV beam were validated at two ProBeam facilities: the Emory Proton Therapy Center multi-room gantry and the Proton International at UAB single-room gantry. The simulations were found to overestimate neutron dose and dose rate by approximately 20% for the multi-room configuration and 18% for the single-room configuration. The simulations calculated the H*(10) ambient dose equivalent using the extended ICRP-74 (20) neutron flux conversions for a 250 MeV proton beam striking a 40x40x40 cm Acrylic water tank with 1 cm thick walls. Results were also compared against radiation safety measurements performed by on-site personnel at Cincinnati Children’s Hospital Medical Center (CCHMC), a multi-room ProBeam facility. In that case, the simulations overestimated dose by about 24% in instances where a calculable dose above background could be obtained. Across all sites, measurements near background level, such as those observed at Cincinnati Children’s Hospital Medical Center (21), produced larger discrepancies relative to on-site readings. Comparisons between simulations and real-world measurements for conventional beam delivery configurations have not yet been completed.

## 5 Detector choice

For secondary photon dose-rate measurements, a range of commercially available devices may be employed, including the Thermo FH-40G-L-10 wide range multi-purpose proportional counter and the Ludlum 9DP ion chamber survey meter. When selecting an appropriate survey meter, it is important to consider that instrument sensitivity can vary considerably depending on the radiation type, dose, and dose rate encountered. For neutron measurements, all contributing authors used the Wide Energy Neutron Detector (version 2), commonly referred to as the Wendi-2, whose specifications are detailed in **Table 1**.

**Table 1:** Wendi-2 neutron detector specifications taken from http://thermofisher.com.

Attribute	Value
Measuring range	0.01 μSv/h to 100 mSv/h Cf-252
Sensitivity	0.84 cps/(μSv/h) Cf-252
Gamma-sensitivity	1 to 5 μSv/h at 100 mSv/h, 662 keV
Energy range	25 meV to 5 GeV according to ICRP 74 (1996)
Angular dependence	± 20% all directions
Ambient temperature	-30 to +50 °C
Humidity	up to 90% non-condensing
Atmospheric pressure	500 to 1500 hPa
Linearity	± 20% Height 320 mm (12.6”)
Diameter	230 mm (9”)
Weight	13.5 kg (29.8 lb)

The Wendi-2 detector is typically calibrated using a ^252^Cf source, which has a 3.09% probability of a neutron-producing decay (22). These neutrons have an energy range from thermal neutrons to 13 MeV, with an average energy of 2.3 MeV and a median energy of 1 MeV. This decay aligns well with the Varian MCNP 250 MeV neutron simulations where 90% of the neutrons generated are below 10 MeV, and the median neutron energy is about 0.2 MeV. From testing, the Wendi-2 neutron detectors used by Varian begin to saturate and produce decreased dose readings at around 15 nA of beam delivery (21). This apparent saturation is present even when the measured neutron dose is below the manufacturer’s saturation limit of 100 mSv/h and it appears to be independent of delivered field characteristics. This saturation may be due to the overall larger neutron flux generated with higher beam currents, but this would need to be investigated further and is outside the scope of this document. The saturation of the Wendi-2 detector was also observed during in-room measurements at the Manchester research facility. **Figure 5** shows the results of neutron dose rate measurements, as a function of nozzle current, using both a ^3^He based Wendi-2 and a BF_3_ based Lupin detector. The measurements were performed with the detectors located 2 meters, perpendicular to the beam direction, away from a PTW plotting tank which was situated 96 cm from the nozzle’s multi-strip ionization chamber (MSIC). The detectors were set to record the maximum dose rate for a 244 MeV proton beam irradiating the water tank for 10 seconds. The saturation of the Wendi-2 detector at 100 mSv/h is evident compared to the Lupin and highlights the need to choose the correct detector for the measurement being undertaken. Detector linearity can be confirmed through the measurement of maximum dose rates as a function of beam current as shown in **Figure 5**.

**Figure 5:**
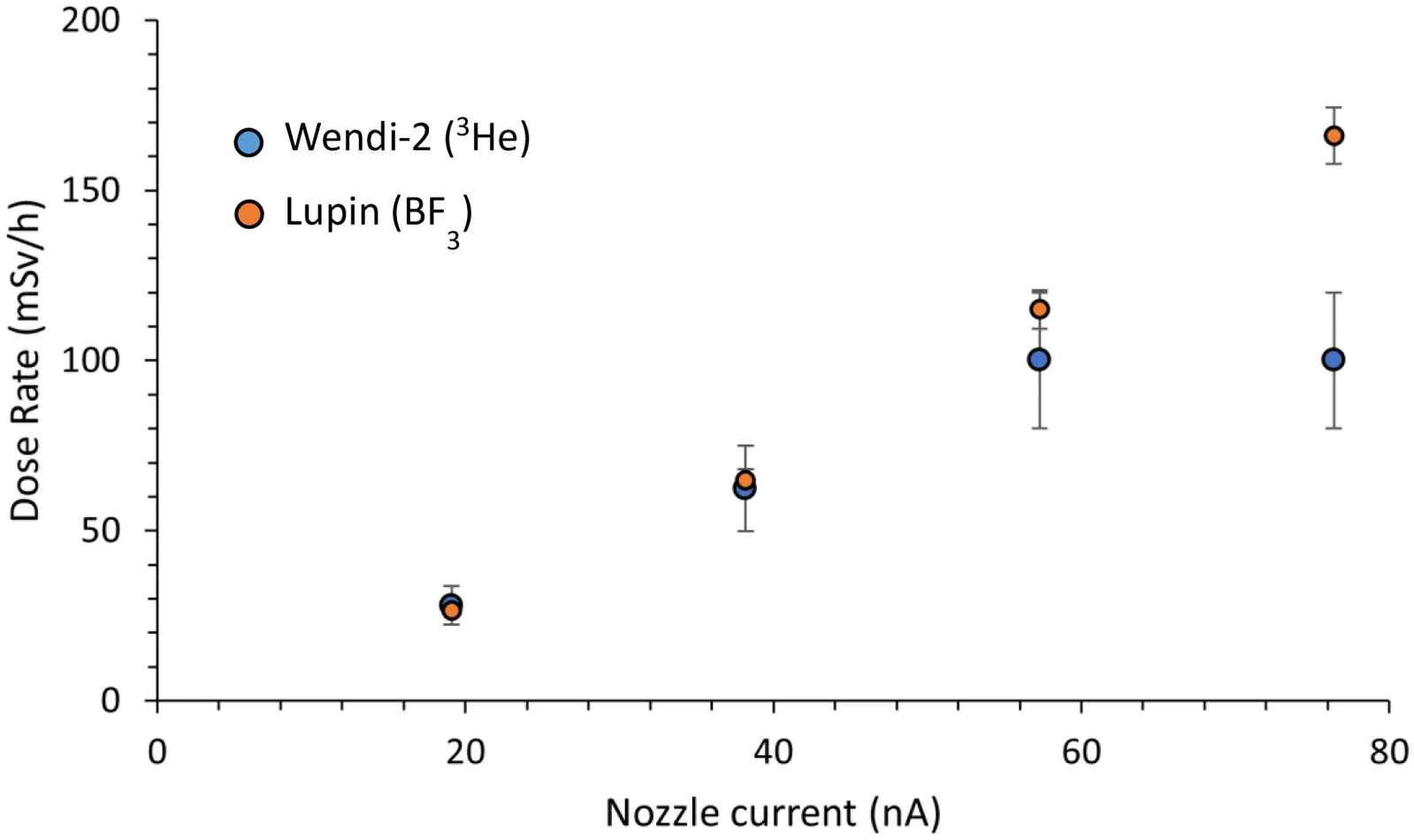
Neutron dose rate measurements, as a function of nozzle current, made with two different detectors, a ^3^He based Wendi-2 and a BF_3_ based Lupin, at the Manchester proton beam therapy research facility. The measurements were performed with the detectors located 2 meters, perpendicular to the beam direction, away from a PTW plotting tank which was situated 96 cm from the nozzle’s multi-strip ionization chamber (MSIC). The detectors were set to record maximum dose rate for a 244 MeV proton beam irradiating the water tank for 10 seconds.

## 6 FLASH dose rate survey

Scaling a conventional dose-rate survey and Monte-Carlo radiation transport simulations can help identify potential areas of concern, however, direct measurements are ultimately required to accurately determine the dose-rates arising from UHDR FLASH beam delivery. Measurements should initially be taken at positions of highest expected dose rate and/or highest expected occupancy to establish if modifications to current controlled areas are required. When planning a survey, consider the location of staff involved in both research and clinical workflows. The survey should include locations like the end of the maze or just outside the vault door, control areas, adjacent rooms, corridors, offices, and relevant 3D locations (e.g. above and below the vaults). Depending on the jurisdiction, in-room measurements may be required to assess responses to accident scenarios.

The beam-on time must be long enough for detector readings to stabilize (a few seconds) for accurate quantitative measurements, which rules out the use of clinical FLASH beams for neutron surveys. For a reasonably accurate survey, one could measure close to the FLASH beam energy, but at lower beam currents, and then scale the results up to the FLASH current. To determine an appropriate scaling factor, measurements should be taken at multiple beam currents, such as 200, 400, 600 and 800 nA (maximum) cyclotron currents. It is important to note that single-spot proton beams can achieve UHDR conditions (> 40 Gy/s) at the center of the spot for beams of 245 MeV and 800 nA cyclotron current. For the actual survey, use the largest current that still produces a linear response from the detector to minimize uncertainty. This approach helps ensure safe working practices during the survey.

Measurements should be repeated to reduce statistical uncertainties and may detect different effects with higher energies and beam currents. Dose rates can change significantly over short distances, so surveys should include enough measurement positions to thoroughly cover the area and highlight hot spots (**Figures 6**, **7**). As emphasized in section 4, it is crucial to use a suitable detector ideally positioned at torso height. Due to the higher dose rates under UHDR conditions, extra radiation safety precautions should be taken when making measurements. Our experience suggests that radiation surveys can quickly accumulate many monitor units. We recommend rapid communication between the surveyor and the beam operator, along with careful tracking and recording of the number of monitor units delivered.

**Figure 6:**
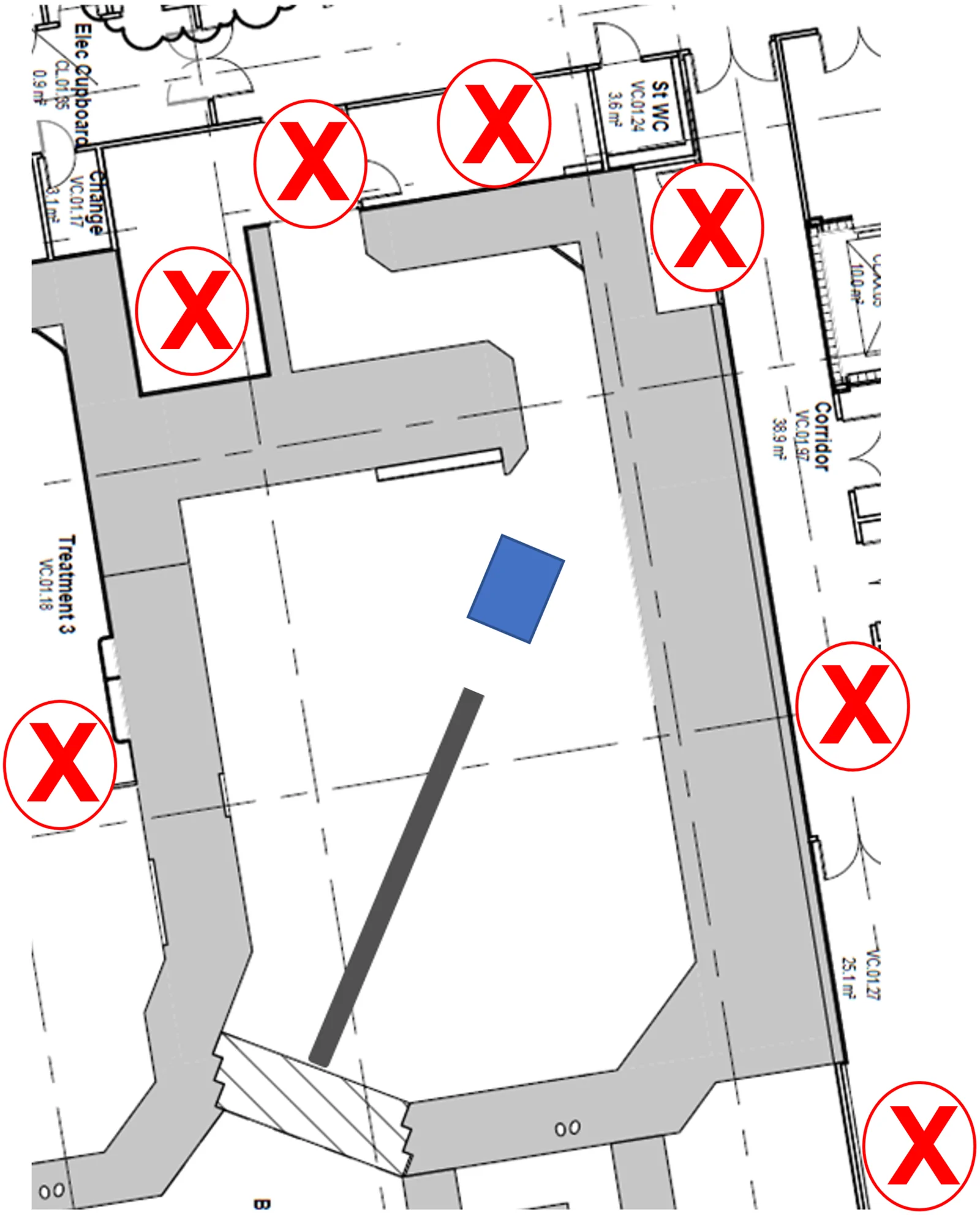
FLASH survey measurement locations (X centered circles) adjacent to the Manchester research room. The full survey covered many more positions spanning four floors, but this is indicative of the locations of potentially high dose rate and/or high occupancy adjacent to the radiation source. The long blue rectangle represents the horizontal beam line and the blue square a PTW water filled plotting tank.

**Figure 7:**
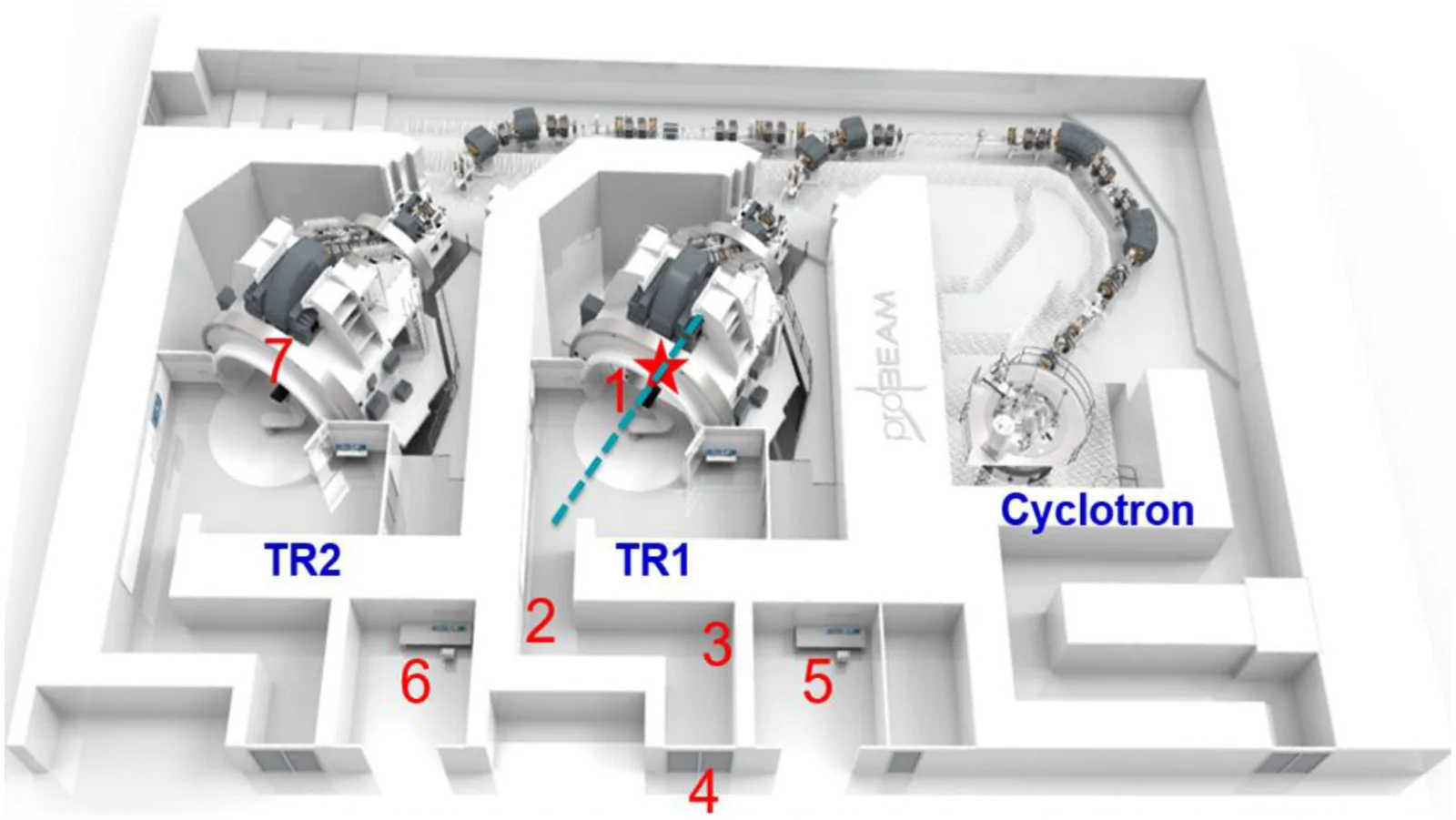
Suggested FLASH survey measurement locations (numbered 1-7) for a ProBeam multi-gantry room (21).

The set up for the dose rate survey should mirror the actual beam usage in practice, as these conditions will influence the effective neutron source. The main neutron source will likely occupy one of two positions: for transmission beams, neutron production occurs at isocenter and primarily at the beam stop; for Bragg peak irradiation, more interactions occur near the nozzle, with the remainder at isocenter. If UHDR is used at multiple gantry angles, the effective neutron source will shift as the gantry rotates, and the impact of rotation should be assessed. At minimum, a “worst case” scenario should be considered. It is important to note that using a water tank or large phantom at isocenter will likely shift the effective location of the neutron source, potentially failing to accurately simulate the actual use of FLASH transmission beams.

For scanned proton UHDR beams, increasing the field size decreases the voxel-averaged dose rate to the target when using the pencil-beam scanning formalism, (3), but this also introduces some variation in the radial fluence. Beam current is the primary driver for neutron dose rates, and changes in proton field size that result in variations in the magnitude of beam deflection are not expected to significantly influence neutron dose rates.

## 7 Beam stops and modulators

In addition to the elevated radiation dose rates present when the beam is operated in UHDR conditions, there may be additional radiation safety challenges introduced in the clinical and research environments, especially with specific beam modulators and beam stoppers used for FLASH delivery in Bragg peak and transmission delivery modes. For transmission beam delivery, the beam passes through the target, delivering a large portion of the dose beyond it. In a research room, a beam stop is recommended to absorb the remaining beam exiting the sample to prevent damage to electronics. In clinical rooms, the use of beam stops may also be feasible, depending on the specific geometry. The beam stop should be designed to fully absorb the proton beam, with careful selection of materials to limit activation. A practical and cost-effective option is a water beam stop, which can be implemented using distilled water in jugs or water tanks (see **Figure 8**), however, it is important to consider that the water containers themselves may become activated and may have a longer half-life than the activated water. Preliminary testing of the beam stops should include measurements of the decay profile at the doses planned for experimental exposure. For the Manchester water-based beam stop shown in **Figure 8**, activity measurements on the order of 3 MBq, 50 seconds after beam off, were recorded for a 0.5 second irradiation with a 244 MeV, 51.1 nA (nozzle current) proton beam. The decay profile will predominantly follow that of ^15^O which has a 2 minute half-life. The beam stop will reach background levels after around 40 minutes.

**Figure 8:**
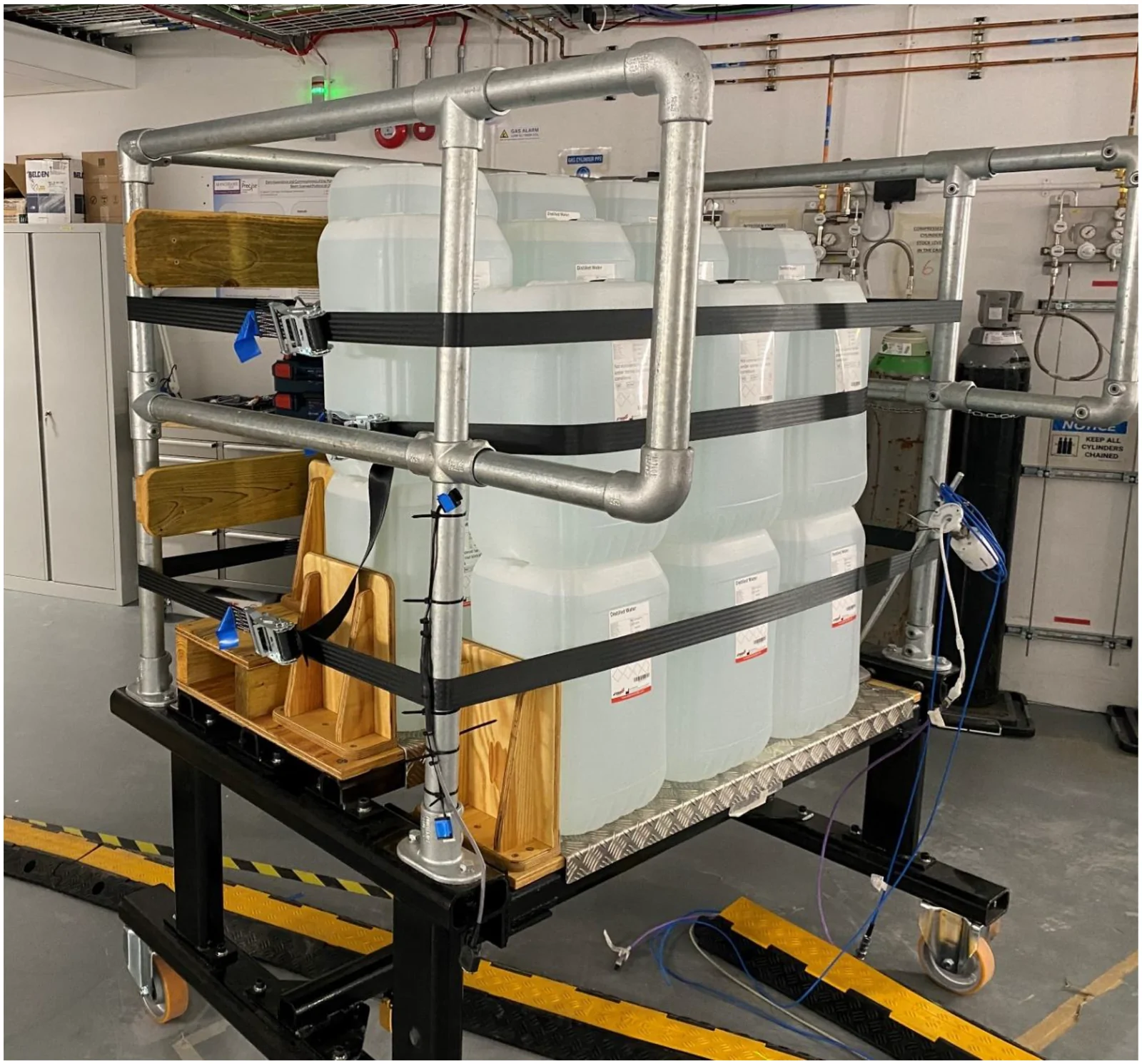
The water-based beam stop used at the Manchester proton beam therapy research center.

Beam stops, acting in a shielding capacity, should be positioned downstream from the experimental setup, as far from the samples as practical, while still covering the entire scanned field with an appropriate safety margin. Beam stops, by their very nature, become radioactive after exposure. Maximizing the distance between the beam stop and the experimental/working area is key to minimizing radiation risk to personnel from beta and gamma radiation. Alternatives to water-based beam stops include high density foam interlocking bricks, while brass has been used for apertures in conventional proton beams. In cases where no beam stops are used, activation measurements of the wall or structure absorbing the beam will be required.

## 8 Activation

The high therapeutic dose rates arising from FLASH beams lead to enhanced activation of elements within the irradiation area from direct interaction with the proton beam. The short beam-on time means less time for these activated elements to decay whilst being irradiated. Also, pre-clinical experiments and hypo-fractionated deliveries require higher than conventionally prescribed doses, leading to higher activation levels. The use of additional material in the beam room, such as beam stops, beam modulating devices and collimators that may be specific to FLASH work, presents a different radiation environment than that commissioned for conventional proton therapy. A radiation protection program should consider the impact of in-room activation and the risk it poses to staff, samples and potential patients. Research samples may become activated to a level that depends on their composition, size and exposure time. Materials comprising copper are of primary importance, due to the combination of ubiquity in proton rooms (brass being a common material used to shape and shield proton beams), short half-life (582 seconds), and high activation cross-section of its primary activated isotope, ^62^Cu. (23).

For example, a brass collimator was designed and optimized to produce six separate irradiation fields with sharper penumbra than those achievable using PBS alone (24). The collimator was carved out of a solid 3 × 3 × 12 in^3^ (7.62 × 7.62 × 30.48 cm^3^) block of brass (stopping ratio 5.75 vs water) with sufficient thickness (~44 cm water-equivalent thickness) to stop the 250 MeV protons used for UHDR irradiations (**Figure 9** (left)). A radiation survey of the activation of the block was performed to ensure staff positioning animals for pre-clinical experimentation would not exceed federal dose limits. While it is impractical to ever exceed the annual dose limit (50 mSv in the US and 20 mSv in the UK), the short-term limit <20 µSv in any one hour is a potential concern. The activation of the collimator block following radiation delivery (17.5 Gy per aperture) was measured every minute in the first 8 minutes and every 5 minutes after 10 minutes at 30 and 5 cm from the collimator.

**Figure 9:**
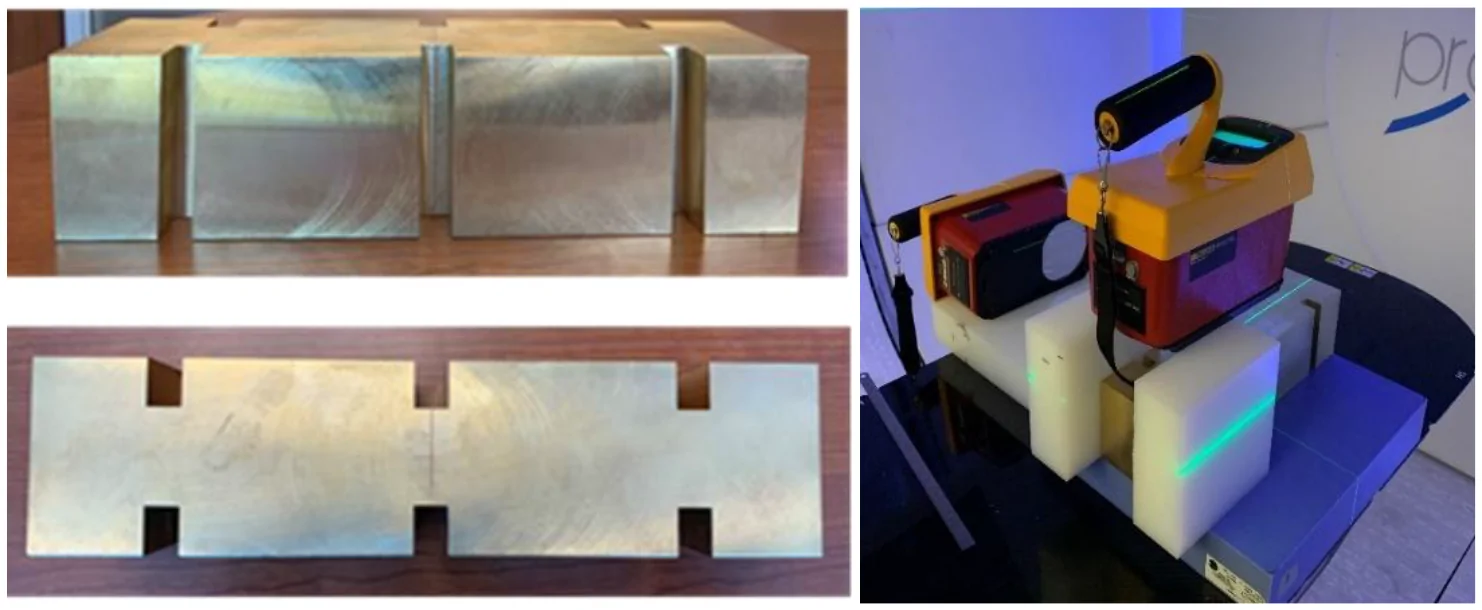
(Left) focused collimator design with six apertures. (Right) Radiation survey setup of activated collimator block following radiation delivery at 5 and 30 cm from the collimator. Effective dose results are shown in **Figure 10**.

**Figure 10** (left) shows the dose rate measurements, at a distance of 5 and 30 cm from the brass collimator to represent extremity or core body dose respectively. **Figure 10** (right) shows the effective dose rate from repeated UHDR delivery allowing for different delay times between deliveries. Depending on the frequency between radiation deliveries, a maximum dose rate plateau is eventually reached after about 60 minutes and ranges from 4.45 to 7.65 µSv/min. Assuming a 5-minute wait before entering the room, the effective dose rate to the whole body at ~30 cm from the collimator reaches 5.4 μSv/min for 10 minutes between deliveries to 3.6 μSv/min for 30 minutes between deliveries. A 20 μSv per any 1 hour limit therefore imposes a 4 minute time limit in proximity (<30 cm) to the collimator. By imposing a 5 minute post-irradiation wait before staff can enter the room, and by devising a radiation platform where mice can be slid in underneath the collimator while retaining the accuracy of the dose delivery, the presence of investigators within <1 m to the activated collimator is kept below one minute (~10 s per animal group), and the total effective dose rate well within the 20 μSv limit. (24).

**Figure 10:**
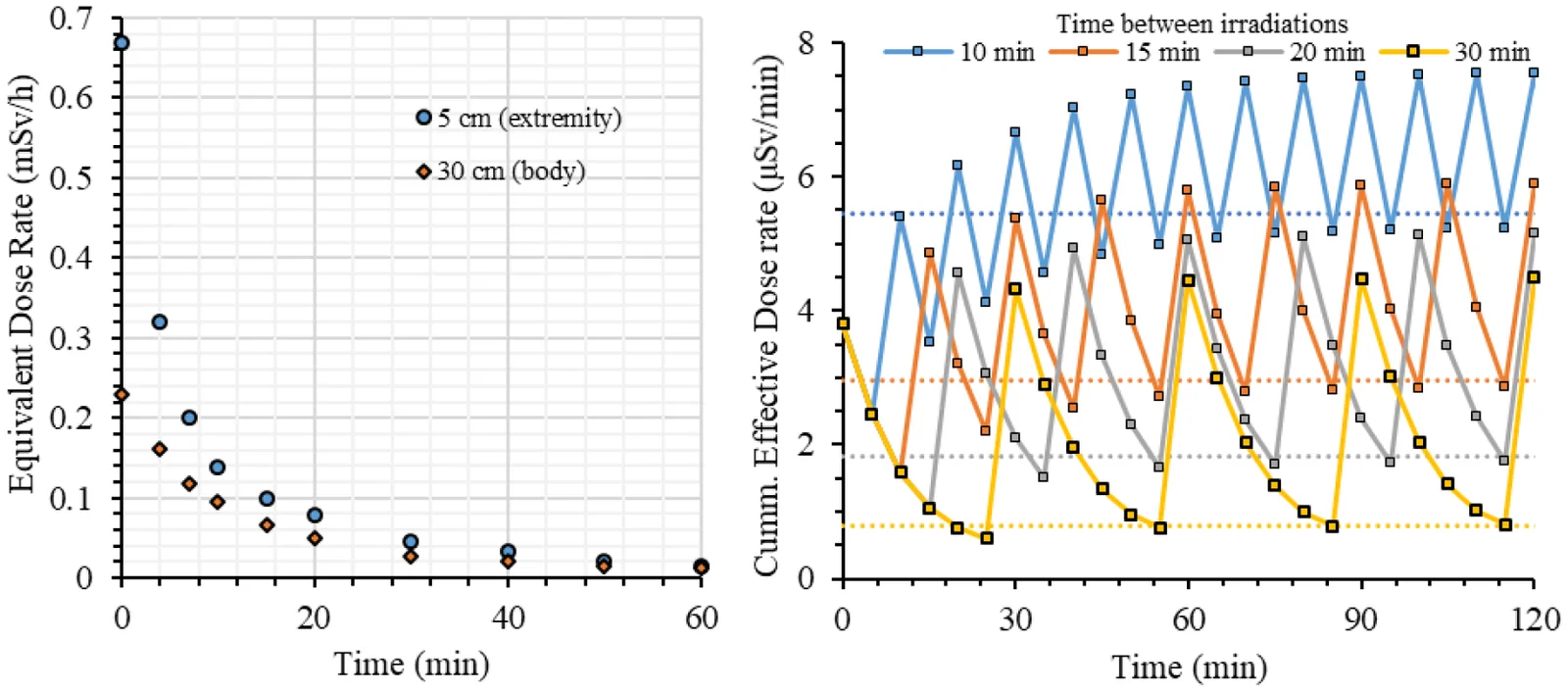
Left: equivalent dose rate measurements (exposure rate and cumulative exposure) of the activated collimator block depicted in **Figure 9** following radiation delivery of 245 MeV protons at 60 Gy/s average dose rate and 17.5 Gy per aperture. Dose equivalent was measured both at 5 and 30 cm from the collimator to represent extremity and whole-body dose of an adjacent staff member. Equivalent dose rate is reported for the extremity (5 cm), while effective dose rate is reported for the whole body (30 cm), assuming gamma decay. Right) Effective dose rate from repeated UHDR delivery allowing for different delay times between deliveries. Shorter delays between repeated deliveries (simulating repeated experiments) allows less decay and increases the equilibrium effective dose rate reached. A maximum effective dose rate is achieved after ~60 minutes regardless of the delay time between deliveries, and ranges from 4.45 to 7.65 µSv/min. Figure reproduced from (24) with permission.

### 8.1 Measures taken to control activation

At FLASH dose rates, the high production of neutrons may also impact in-room silicon-based electronics. This may include dosimetry, personal protection system and experimental equipment such as computers and electrometers and even electronics for the beam delivery system. It should first be considered whether it is necessary for the electronic equipment to be in the room if portable and if so, suitable neutron shielding would be advised. One possible shielding solution is the use of boronated polyethylene sheets which have excellent thermal neutron capture properties, thus shielding sensitive electronic components from potential neutron damage. If a patient or staff member in the vicinity has a pacemaker, caution is urged to reduce as much as possible the potential neutron dose.

When entering an irradiation area shortly after a beam-on period, it is recommended that personnel should wear an electronic personal dosimeter (EPD) whose alarm level can be set to the local acceptable dose rate level. It is also recommended to use a hand-held survey meter to ascertain the activation of equipment in the vicinity of the work area or of items that need to be handled. It is important to survey all apparatus that may have been irradiated by the primary beam e.g. couch and surrounding infrastructure. Research equipment should be safely stored in a designated radioactive decay storage area or left with clear signage *in-situ* if significant activation is detected.

When utilizing a beam stop, a water/plastic-based system is best due to the relatively short half-lives of the activated isotopes (~2 min for water and 20 min for general hydro-carbons). Although the half-lives of the most commonly activated isotopes are relatively short, a water-based beam stop can become highly activated when using FLASH beams. This poses a radiation hazard for staff conducting research in the vicinity of the beam stop. A radiation monitor should be used to survey the beam stop before work is undertaken in its vicinity. The main radiation risk arises from the 511 keV photons produced in the positron annihilation process from the decay of isotopes such as ^15^O and ^11^C (although other beta-delayed gamma-rays should also be considered). The location of the beam stop could be critical because it is both a source of neutrons and photons when the beam is on, generated by proton inelastic reactions, but also an activation risk when the beam is off. Extra shielding requirements may be needed depending on its position.

At the Manchester center a sampling handling limit of 75 μSv/h measured at the sample surface is imposed, with the use of tongs and gloves recommended for samples producing instantaneous dose-rates in excess of 10 μSv/h. These limits are locally imposed at the Manchester center and are based on the ALARP (As Low As Reasonably Practicable) principle in order to keep staff safe but not restrict crucial research. Each center should adopt limits in accordance with its own national and local regulations and guidelines. The time to safe handling will depend on the amount of activation which, in itself, is dependent on beam energy, current, and irradiation time. All samples should be measured prior to handling and at the Manchester center, passive ring dosimeters are provided to experimenters who could be handling activated samples or hardware to record the dose received to the extremities.

**Table 2** summarises the dominant radioactive isotopes produced through the activation of common materials used in proton FLASH research along with the corresponding decay half-lives. It is clear from the stated, relatively short, half-lives why water is the chosen material for a beam stop with plastic as the container for the water and brass is typically used for collimators. Material choices should be a balance between decay time, structural integrity and fit-for-purpose.

**Table 2:** Summary of the dominant radioactive isotopes produced through the activation of common materials used in proton FLASH research with their corresponding decay half-lives.

Material	Dominant isotopes	Half-life
Water	^15^O	122 s
Plastic	^11^C	20.4 min
Brass	^62^Cu, ^63^Zn	9.7 m, 38.1 m
Aluminium	^26^Al^m^	6.3 s
Copper	^62^Cu	9.7 m
Steel	^55^Fe	2.7 y
Tungsten	^185^W	75.1 d

Air in the irradiation area may also become activated at a rate not fully compensated for by decay or air exchanges. A simple precaution is to install a wall monitor to warn staff if the dose rate in the room exceeds a set limit. **Figure 11** shows the wall mounted ionisation chamber used to measure air activation within the irradiation area at the Manchester facility. The detector is coupled to a digital readout display near the maze entrance with a warning light that is set to illiminate when the ambient dose rate exceeds 7.5 µSv/h. A wall mounted detector can monitor in-room activation and has the added benefit of indicating the presence of beam due its sensitivity to charged and neutral particles. This independent beam-on sensitivity is an added protection in case of a complete system malfunction. Some activity monitoring systems have a recording option, which is useful for estimating the dose received in an accident scenario, e.g. a person is accidentally present in the room during a delivery.

**Figure 11:**
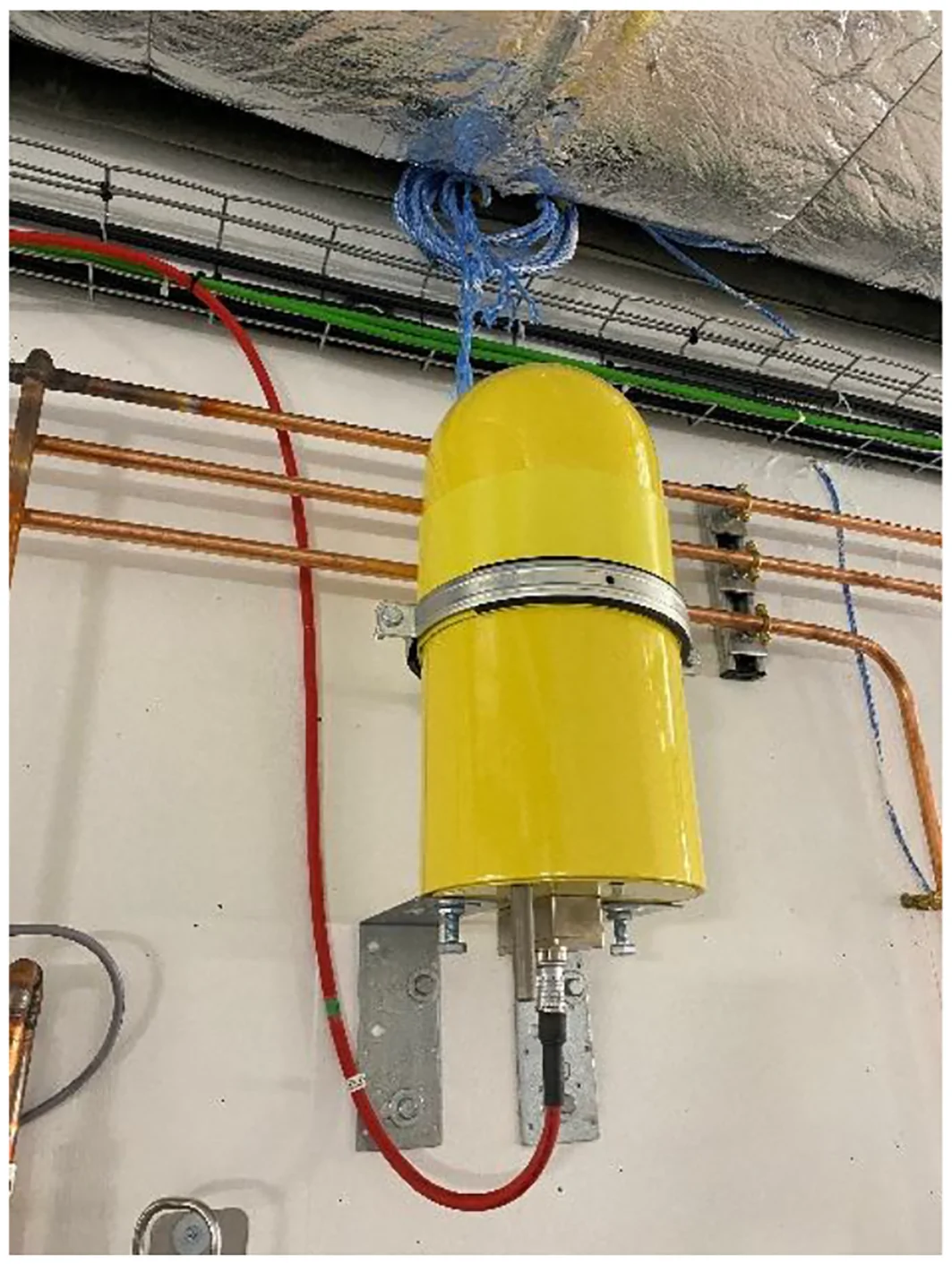
An example of a wall mounted ionization chamber type detector, used at the Manchester center, for monitoring activated air in the irradiation area.

## 9 Accident scenarios

The increased dose rate from FLASH beams presents an enhanced hazard in the beam delivery room as large doses of radiation may be delivered in a short period of time. For this reason, it is important to implement process controls and checks to prevent extreme doses to personnel being delivered. Following the last person out procedure when securing the treatment room for FLASH delivery is critically important in preventing accidents. The manufacturer, Varian, has set limits for the number of monitor units (MU) that can delivered in a certain time to protect the proton therapy delivery equipment itself. The radiation safety program may wish to impose stricter limits to protect staff and/or the public.

The Manchester centre has adopted a two-person room securing and control room person count protocol to minimize the risk of an experimenter accidentally remaining in the controlled area prior to a beam on request. There is a no lone working policy in place ensuring at least two experimenters are available to search the controlled area before activating the personnel protection system. Following the closure of the controlled area gate, all experimenters must be accounted for and present in the beam control room prior to any beam request. Experimenters rehearse emergency procedures for in room accidents as part of the radiation safety training. In the event that an experimenter does accidentally remain within the controlled area and the beam is delivered, the estimated absorbed dose would be around 28 mSv based on the results in **Figure 5** and a beam on time of 10 seconds; a suitable length of time for an experimenter to realize the beam is on and be able to hit an emergency beam off button, of which there are normally several, in the controlled area.

The personnel protection system, in most centers, has a beam interruption function should an experimenter try to enter the controlled area whilst the beam is on. This is via an interlock on the physical barrier to the controlled area, usually in the form of a latch or microswitch. This beam interrupt should be tested on a regular basis, usually as part of a general radiation protection audit, but could be tested prior to any session where FLASH beams may be delivered. Likewise, regular checks for any warning lights and area monitors should form part of a general radiation protection audit but could be checked before any FLASH beams are to be delivered. The main radiation risks for experimenters entering the controlled area shortly after irradiation arise from activation which is covered in section 7.1.

For facilities that utilize a beam stop, in order to ensure the beam stop is in place and has not accidentally been moved or removed, an engineering solution should be sought to ensure its presence prior to the delivery of FLASH beams. The beam stop in the Manchester center is interlocked via a microswitch to the beam request system. This ensures that the beam stop is in the correct location prior to any beam delivery to the controlled area as a beam request cannot be submitted if this interlock is broken by the non-presence of the beam stop. The water is resides in sealed plastic containers ensuring that sufficient water is always present to stop 250 MeV proton beams.

**Table 3** summarizes potential accident scenarios, associated risks, conventional beam delivery control measures and additional FLASH beam delivery control measures.

**Table 3:** A summary of potential accident scenarios, the associated risks, the conventional beam delivery control measures along with any additional FLASH beam related control measures, applied by the centers featured in this work.

Accident scenario	Risks	Existing controls	Additional FLASH controls
Experimenter in controlled area whilst beam is on	Radiation exposure from the primary proton beam and secondary particles (neutrons and gammas)	Last person out procedure for activating the personnel protection system and securing the area for beam delivery Emergency beam shut off buttons within and outside of the controlled area Emergency scenario rehearsals as part of the general radiation safety training	Area sweep done by two experimenters and a head count outside of the controlled area prior to any beam request
Experimenter entering the controlled area whilst the beam is on	Radiation exposure from the primary proton beam and secondary particles (neutrons and gammas)	Beam interruption through an interlock break in the personnel protection system activated by the sensor on the physical barrier at the entrance of the controlled area Periodic checks of the interlock beam interruption mechanism as part of a standard radiation protection audit	Personnel protection system beam interruption tests conducted immediately prior to a FLASH beam delivery session.
Warning lights or monitor failure	Radiation exposure from the primary proton beam and secondary particles (neutrons and gammas) if within the controlled area or from activated components if entering the controlled area	Warning lights and monitors are periodically checked as part of a standard radiation protection audit with monitors calibrated annually via a certified service	Warning lights and monitors physically checked immediately before a FLASH beam delivery session.
Beam stop not in correct position or removed completely	Risk of radiation exposure outside of the controlled area	Beam stop is interlocked to the beam request system	Observational check that the beam stop is in its correct position

## 10 Discussion

Assessing current radiation protections and instigating new provisions for proton FLASH research is a significant undertaking. For many PBT centers this will be a new endeavor and a steep learning curve will need to be climbed. During the investigations outlined in this document, many lessons were learned with the most notable being discussed in this section. It should be emphasized that this work does not make recommendations but merely documents the lessons learnt by members of the FLASH forward consortium radiation protection working group whilst undertaking the detailed FLASH radiation protection investigations. Centers looking to perform FLASH research should follow their countries legislative framework for radiation protection. It may be useful to separate the lessons learned by type of center where FLASH research is being undertaken. Research undertaken using a clinical gantry poses a differing set of challenges to that of a dedicated research facility.

### 10.1 Clinical gantries

FLASH radiation protection studies conducted at Cincinnati Children’s Hospital Medical Center (CCHMC) highlighted the challenges surrounding dose-rate surveys, particularly the measurement of neutron dose rates with commercially available neutron dosimeters (21). They noted that an off-the-shelf neutron dosimeter, with its relatively slow time constant, is at odds with the fast response required for UHDR measurements. It is important to understand the measurement limitations and uncertainty associated with survey devices to ensure the measurement data is reliable. They also highlighted that careful consideration of the effort to conduct radiation protection research in a clinical center is required, specifically when those staff members have clinical duties. During the dose-rate survey at CCHMC it was noted that the highest ambient dose rate of 2.46 mSv/year, approximately 5% of the maximum permissible (US regulations) annual occupational dose, was identified at the isocenter of the adjacent treatment room with a 90° gantry angle used for transmission FLASH beam delivery without a beam stop. Beam angle is a key consideration when conducting FLASH research using a clinical gantry due to the potentially high dose rate fields that may arise in a neighboring treatment room. The effect on a patient in the adjacent room can be reduced by controlling the use factor in the adjacent room while FLASH beams are being delivered. The investigations reported in this work are transferable to other cyclotron-gantry based systems but the results presented will be specific to the center they pertain to. Each clinical center should conduct its own radiation protection investigations as the maximum beam energy, cyclotron current and beam transport may differ from those of a Varian center.

### 10.2 Dedicated research facilities

From a research facility perspective, one lesson learned in particular was that a full center survey should be undertaken as areas of higher-than-expected dose-rates can exist which may not appear obvious based on beam line geometry and expected shielding; of note are areas above and below the irradiation area. Monte-Carlo radiation transport simulations predicted an ambient neutron dose rate of 0.2 μSv/h in the plant room above the maze in the Manchester center whereas the survey yielded a dose rate 265 μSv/h. Beam stops play an important role in attenuating the primary beam following a sample irradiation, but it is important to understand the implications and positioning of a beam stop as it can become a source of neutrons which also poses a radiation risk.

### 10.3 General considerations

Key considerations that span both clinical and research facilities include the use of Monte Carlo radiation transport codes to indicate potential high-risk areas. Simulations need to be validated, and it is important to ensure that virtual geometries reflect the real geometry in both dimensions and material compositions. Measuring neutron fields is a not trivial task, even more so considering the wide range of neutron fluences and energies produced during high-energy proton beam therapy. Neutron detectors should be chosen with this in mind, and independent repeated measurements should be performed if possible. Proton beam therapy centers by their very nature are large, with multiple layers of shielding. Conducting FLASH dose rate surveys can be time consuming as repeated measurements are required. The survey time is extended further when measuring in areas of expected high dose rates as measurements as a function of increasing beam current may be needed to protect the staff undertaking the survey.

## 11 Conclusion

The work described in this paper has enabled FLASH research to be conducted both safely and legally in several Varian ProBeam facilities, worldwide. The novelty of proton FLASH beams has meant that centers have had to conduct research into the radiation protection provisions required for FLASH delivery. Radiation protection research may receive less attention than biological or clinical aspects of FLASH radiotherapy, but it is essential for the safe development of novel treatment techniques. The work detailed is not exhaustive but provides insight into the issues surrounding radiation protection of UHDR proton beams for both existing centers and those planning to commence proton FLASH research. Several considerations emerge from this work, however, centers should always consult and follow the advice of their legally appointed radiation protection advisor.

## Data Availability

The datasets presented in this article are not readily available due to commercial sensitivity but some aspects could be made available with permission. Requests to access the datasets should be directed to wendy.smith@varian.com.
